# Spatial–Spectral Constrained Adaptive Graph for Hyperspectral Image Clustering

**DOI:** 10.3390/s22155906

**Published:** 2022-08-07

**Authors:** Xing-Hui Zhu, Yi Zhou, Meng-Long Yang, Yang-Jun Deng

**Affiliations:** 1 College of Information and Intelligence, Hunan Agricultural University, Changsha 410128, China; 2 Hunan Provincial Engineering and Technology Research Center for Rural and Agricultural Informatization, Hunan Agricultural University, Changsha 410128, China

**Keywords:** clustering, adaptive graph, spatial–spectral constraint, hyperspectral image

## Abstract

Hyperspectral image (HSI) clustering is a challenging task, whose purpose is to assign each pixel to a corresponding cluster. The high-dimensionality and noise corruption are two main problems that limit the performance of HSI clustering. To address those problems, this paper proposes a projected clustering with a spatial–spectral constrained adaptive graph (PCSSCAG) method for HSI clustering. PCSSCAG first constructs an adaptive adjacency graph to capture the accurate local geometric structure of HSI data adaptively. Then, a spatial–spectral constraint is employed to simultaneously explore the spatial and spectral information for reducing the negative influence on graph construction caused by noise. Finally, projection learning is integrated into the spatial–spectral constrained adaptive graph construction for reducing the redundancy and alleviating the computational cost. In addition, an alternating iteration algorithm is designed to solve the proposed model, and its computational complexity is theoretically analyzed. Experiments on two different scales of HSI datasets are conducted to evaluate the performance of PCSSCAG. The associated experimental results demonstrate the superiority of the proposed method for HSI clustering.

## 1. Introduction

Hyperspectral remote sensing combines imaging and spectral technologies together to detect objects remotely. The resulting hyperspectral images (HSIs) contain rich spatial and spectral information, which are able to distinguish objects with small dissimilarity. Therefore, HSIs have been widely used in various fields [1,2], such as agriculture [3], urban planning [4], environment monitoring [5], etc. In these applications, HSIs play an important role in the classification of ground objects, which is often achieved by hyperspectral image (HSI) segmentation. Currently, the HSI segmentation methods can be roughly divided into two categories: supervised and unsupervised ones. Supervised HSI segmentation is generally known as HSI classification, whose representative methods include support vector machine (SVM) [6], sparse representation-based classifier (SRC) [7], extreme learning machine (ELM) [8], and so on. Nevertheless, HSI classification requires a lot of well-labeled samples to train the model [9], which limits its application. For unsupervised HSI segmentation, the HSI labeling is unnecessary, which can greatly simplify the data processing. As the most important part of the unsupervised HSI segmentation, HSI clustering attracts extensive attention due to its simplicity and efficiency. To this end, this paper mainly focuses on HSI clustering.

In the past decades, numerous clustering methods have been proposed and applied for HSI segmentation, among which K-means clustering is one of the most classical clustering algorithms [10]. It converges quickly and generally performs well on small-scale data. However, K-means clustering is sensitive to noise and prone to falling into the local optimum. Furthermore, it cannot handle non-convex data. To overcome those shortcomings, the basic iterative self-organizing data analysis algorithm (ISODATA) was proposed to improve the K-means clustering [11], where the parameter *K* is updated in each iteration. The non-negative matrix factorization (NMF) was developed by factorizing data matrix into two low-dimensional non-negative factor matrices to achieve less computational cost on HSI clustering [12]. Fuzzy c-means clustering (FCM) was proposed to avoid the hard-clustering deficiency by utilizing the fuzzy membership [13]. It achieves clustering by calculating the membership of each sample to all classes, where the value of membership is between 0 and 1. However, FCM often produces clustering maps with salt and pepper noise. Considering the spatial continuity of objects, the spatial constraint was applied to exploit the spatial information of image data for enhancing the robustness of FCM, resulting in the fuzzy clustering with spatial constrains (FCM_S) [14]. In order to reduce the complexity of FCM_S, FCM_S1 was proposed [15]. Additionally, by integrating the idea of weighted mean into the FCM, Li. et al. [16] proposed the fuzzy weighted c-means (FWCM) to improve the performance of clustering. Subsequently, a new weighted fuzzy C-means algorithm (NW-FCM) was proposed for solving similar high-dimensional multiclass pattern recognition problems [17]. However, these extended FCM algorithms need a parameter to control the balance between robustness to noise and the effectiveness of preserving details; the selection of these parameters is difficult in practice. To overcome the above shortages, Krinidis and Chatzis [18] presented a fuzzy local information c-means (FLICM). In FLICM, the center pixel is greatly affected by its neighboring pixels. Thus, to trade off the center pixel’s own features and the influence of neighboring pixels, a novel adaptive FLICM (ADFLICM) clustering approach was proposed to modify FLICM [19].

The subspace clustering methods generally model the same-class pixels that have various spectral signatures with a subspace and approximate the complex internal structure of HSIs by a union of subspaces, which may relieve the large spectral variability and improve the modeling accuracy [20]. The most representative subspace clustering model is sparse subspace clustering (SSC), which was proposed to group data points into different subspaces by finding the sparsest representation for each data point [21,22]. Combined with the spatial information and the nonlinearity of HSIs, many modified SSC methods have been proposed for HSI clustering. For example, a novel spectral–spatial sparse subspace clustering (S${}^{4}$C) was developed to explore the spectral similarity of local neighborhoods for improving the SSC model by incorporating the wealthy spatial information of HSI [23]. In addition, in [24], a spectral–spatial SSC based on 3D edge-preserving filtering (SSC-3DEPF) algorithm was put forward. It utilizes 3D edge-preserving filtering for the sparse coefficient matrix obtained by SSC to extract the spectral–spatial information to generate a more accurate coefficient matrix, which is favorable for clustering. A joint SSC (JSSC) method [25] was proposed to make use of the spatial information through joint sparse representation. It forces the pixels in a spatial neighborhood to share the same sparse basis. As the advantages of deep structures have been extensively verified, SSC also was extended to deep vision. To make full use of spatial information, a novel spectral–spatial Laplacian regularized deep subspace clustering (LRDSC) algorithm is proposed for HSI analysis [26]. Furthermore, a novel deep spatial–spectral subspace clustering network (DS${}^{3}$C-Net) is proposed to learn the similarity relationship among the pixels for improving HSI clustering [27].

Another important kind of clustering technique is the recent graph-based approaches. The main idea of these methods is to make the similarities within the sub-graphs as high as possible while making the edge weights connecting different sub-graphs as low as possible. The typical ones include clustering with adaptive neighbors (CAN) [28], projected CAN (PCAN) [28], fast spectral clustering with anchor graph (FSCAG) [29], and a scalable graph-based clustering with non-negative relaxation (SGCNR) [30]. CAN can adaptively acquire the local geometry of the data via constructing an adaptive adjacency graph and greatly improve the accuracy of clustering. The PCAN model attempts to learn a low-dimensional projection for reducing the dimensionality of data while clustering with adaptive neighbors. FSCAG and SGCNR greatly reduce the computational complexity by constructing the anchor graph such that they can be applied for large-scale HSI processing.

Although the previous HSI clustering methods have achieved great success, there are still two questions that need further study in HSI clustering. On the one hand, most of those methods are sensitive to noise corruption and cannot capture the intrinsic structure of data with noise accurately. On the other hand, the high-dimensionality of HSI data not only leads to a huge increase in clustering cost, but also limits the performance of HSI clustering. To tackle the above problems, this paper proposes a projected clustering with a spatial–spectral adaptive graph (PCSSCAG) for HSI segmentation. PCSSCAG first constructs a spatial–spectral constrained adaptive graph with the locality structure adaptive acquisition technique, which can precisely capture the local geometrical structure information of HSI data. Meanwhile, a spatial–spectral constraint is utilized to simultaneously exploit the spatial and spectral information of HSI, which can further suppress the negative impacts of noise to improve the quality of the adaptive adjacency graph. Then, projection learning is integrated into the construction of spatial–spectral constrained adaptive graph to solve the problems that arise from high-dimensional features. Finally, this paper designs an alternating iteration algorithm to optimal the proposed model and theoretically analyze the computational complexity of the optimization algorithm. In summary, the proposed PCSSCAG method can simultaneously exploit spatial–spectral information and adaptively capture the locality geometrical structure to enhance the robustness against noise. Moreover, PCSSCAG can preserve the information reflected by the adaptive adjacency graph in the low-dimensional space to improve the performance of clustering. In addition, the low-dimensional projection, the captured locality structure, and the clustering results will fine-tune each other to obtain better solution at every iteration of the optimization algorithm. Extensive experiments on some benchmark HSI datasets demonstrate the effectiveness of the proposed method.

## 2. Methodology

This section first introduces, in detail, the formulation of the proposed PCSSCAG model. Then, an alternating iteration algorithm is designed to optimize the proposed model. At last, the complexity of the optimization algorithm is theoretically analyzed, and a parallel computation strategy of PCSSCAG is proposed for large-scale HSI clustering.

### 2.1. Formulation of PCSSCAG

In general, due to the influences of imaging environments and the characteristics of the imaging system, the obtained HSI data are inevitably disturbed by noise, which seriously degrades the quality of the data and limits the performance of HSI clustering. For the graph-based clustering methods, the quality of the adjacency graph plays an important role in the clustering. The more accurate the local geometrical structure captured by the adjacency graph, the better the clustering performance yielded. Thus, how to capture the precise intrinsic structure from the noisy data is critical for improving the accuracy of HSI clustering. Recently, the locality neighbors adaptive acquisition technique has provided an effective choice for characterizing noisy data, which can reveal the intrinsic structure of data adaptively. Inspired by CAN, an adaptive adjacency graph is first constructed to capture the accurate local geometrical structure of HSI data for improving the performance of HSI clustering. In detail, supposing a data matrix $X\in \{x_{1},x_{2},\ldots ,x_{i},\ldots ,x_{n}\}$, *n* is the number of samples. Then, we can deal with the following problem to obtain the adjacency graph for the HSI data

(1) $$\min_{\forall i,s_{i}^{T}1=1,0\leq s_{i}\leq 1}\sum_{i,j=1}^{n}(∥x_{i}-x_{j}{∥}_{2}^{2}s_{ij}+\gamma s_{ij}^{2}),$$

where $x_{i}\in R^{m\times 1}$, $s_{ij}$ represents the similarity between $x_{i}$ and $x_{j}$. $s_{i}\in R^{n\times 1}$, and the *j*-th element is $s_{ij}$. γ is the regularization parameter. It can be known from problem (1) that the smaller distance of $∥x_{i}-x_{j}{∥}_{2}^{2}$ corresponds to the higher probability $s_{ij}$.

Since the local neighbor pixels have a high probability of belonging to the same cluster, spatial information plays a critical role for the segmentation of HSI [31], which can effectively suppress the impact of noise to yield a more smooth segmentation map. However, the problem (1) fails to consider the spatial information. Therefore, in order to construct a more accurate adaptive adjacency graph for HSI clustering, a spatial–spectral constraint term is added to (1) for exploiting the spatial–spectral information to enhance the robustness. Mathematically, the objective function with spatial–spectral constraint is expressed as

(2) $$\begin{array}{c} \min_{\forall i,s_{i}^{T}1=1,0\leq s_{i}\leq 1}\sum_{i,j=1}^{n}\left(\right\|x_{i}-x_{j}{\|}_{2}^{2}s_{ij}+\gamma s_{ij}^{2})+\frac{\beta }{N_{r}}\sum_{i,j=1}^{n}\sum_{k=1}^{N_{r}}{\left\|x_{i}^{k}-x_{j}^{k}\right\|}_{2}^{2}s_{ij}, \end{array}$$

where β is the impact factor of the spatial–spectral constraint, $x_{i}^{k}$ is the *k*-th neighbor pixel of $x_{i}$, and $N_{r}$ is the number of neighbor pixels.

The ideal adjacency graph with a clear clustering structure can be achieved by adding an additional constraint rank(Ls)=n−c into the problem (2). Thus, the new clustering model is to solve

(3) $$\begin{array}{cc} \; & \min_{S}\sum_{i,j=1}^{n}\left(\right\|x_{i}-x_{j}{\|}_{2}^{2}s_{ij}+\gamma s_{ij}^{2})+\frac{\beta }{N_{r}}\sum_{i,j=1}^{n}\sum_{k=1}^{N_{r}}{\left\|x_{i}^{k}-x_{j}^{k}\right\|}_{2}^{2}s_{ij}\\ \; & \forall i,s_{i}^{T}1=1,0\leq s_{i}\leq 1,rank\left(Ls\right)=n-c, \end{array}$$

where $S\in R^{n\times n}$, and the *i*-th element is $s_{i}$. Moreover, Ls is the Laplacian matrix, where $Ls=D-\frac{S^{T}+S}{2}$ and $D=\sum_{j}(s_{ij}+s_{ji})/2$ is a diagonal matrix. According to [18], the problem (3) can be transformed into

(4) $$\begin{array}{cc} \; & \min_{S,F}\sum_{i,j=1}^{n}\left(\right\|x_{i}-x_{j}{\|}_{2}^{2}s_{ij}+\gamma s_{ij}^{2})+\frac{\beta }{N_{r}}\sum_{i,j=1}^{n}\sum_{k=1}^{N_{r}}{\left\|x_{i}^{k}-x_{j}^{k}\right\|}_{2}^{2}s_{ij}+2\lambda Tr\left(F^{T}LsF\right)\\ \; & \forall i,s_{i}^{T}1=1,0\leq s_{i}\leq 1,F\in R^{n\times c},F^{T}F=I, \end{array}$$

where $2\lambda Tr\left(F^{T}LsF\right)=\sum_{i,j=1}^{n}\lambda {∥f_{i}-f_{j}∥}_{2}^{2}s_{ij}$, $f_{i}$ is cluster indicator vector. The smaller the distance of $|f_{i}-f_{j}{∥}_{2}^{2}$, the stronger the similarity. It means that the probability ($s_{ij}$) that two samples belong to the same cluster is greater.

Furthermore, as mentioned in the Introduction, HSI data have the characteristics of high dimensionality, which often contain an amount of redundancy and lead to high computational cost. To address these problems, we integrate projection learning into the above model and develop a method named projected clustering with a spatial–spectral constrained adaptive graph (PCSSCAG) for HSI clustering. The corresponding objective function is formulated as

(5) $$\begin{array}{cc} \; & \min_{S,P,F}\sum_{i,j=1}^{n}(∥P^{T}x_{i}-P^{T}x_{j}{∥}_{2}^{2}s_{ij}+\gamma s_{ij}^{2})+\frac{\beta }{N_{r}}\sum_{i,j=1}^{n}\sum_{k=1}^{N_{r}}{∥P^{T}x_{i}^{k}-P^{T}x_{j}^{k}∥}_{2}^{2}s_{ij}+2\lambda Tr\left(F^{T}LsF\right)\\ \; & \forall i,s_{i}^{T}1=1,0\leq s_{i}\leq 1,P^{T}StP=I,F\in R^{n\times c},F^{T}F=I, \end{array}$$

where $P\in R^{d\times m}\left(d<<m\right)$, and $S_{t}=X^{T}HX$, where $H=I-\frac{1}{n}11^{T}$.

Remarkably, while allowing the projection $P=I\in R^{m\times m}$, the PCSSCAG model will degenerate into (4), which is a special case of PCSSCAG with clustering on the raw data with a spatial–spectral constrained adaptive graph. Thus, to extensively verify the effectiveness of PCSSCAG, we denote the degenerated model as clustering with spatial–spectral constrained adaptive graph (CSSCAG) for comparison in the experimental part.

### 2.2. Optimizayion of PCSSCAG

In the constructed PCSSCAG model, there are three variables (*S*, *P*, and *F*) that need to be solved. It is difficult to obtain the optimal solution for all variables at the same time. Therefore, an alternating iteration algorithm is designed to solve the three variables. Firstly, we initialize S by solving problem (1). Then, the iterative algorithm consists of the following three steps:

(1) Update *F*
  If *S* and *P* are fixed, the optimal *F* can be computed by
  (6) $$\begin{array}{cc} \; & \min_{F\in R^{n\times c},F^{T}F=I}Tr\left(F^{T}LsF\right). \end{array}$$
  The optimal *F* is formed by the *c* eigenvectors of $Ls=Ds-\frac{S^{T}-S}{2}$ corresponding to the *c* smallest eigenvalues.

(2) Update *P*
  Assuming *F* and *S* is given, the optimization problem becomes
  (7) $$\begin{array}{cc} \; & \min_{P^{T}StP=I}\sum_{i,j=1}^{n}(∥P^{T}x_{i}-P^{T}x_{j}{∥}_{2}^{2}s_{ij}+\frac{\beta }{N_{r}}\sum_{i,j=1}^{n}\sum_{k=1}^{N_{r}}{∥P^{T}x_{i}^{k}-P^{T}x_{j}^{k}∥}_{2}^{2}s_{ij}. \end{array}$$
  It can be written as
  (8) $$\begin{array}{cc} \; & \min_{P^{T}StP=I}Tr\left(P^{T}X^{T}LsXP\right)+\beta Tr\left(P^{T}M^{T}LsMP\right), \end{array}$$
  where $M=\frac{1}{N_{r}}\sum_{k=1}^{N_{r}}X^{k}$, and $X^{k}=\left[x_{1}^{k},x_{2}^{k},\ldots ,x_{n}^{k}\right]$. The optimal solution is formed by the m eigenvectors of $St^{-1}X^{T}LsX$ corresponding to the m smallest eigenvalues.

(3) Update *S*
  Due to $2\lambda Tr\left(F^{T}LsF\right)=\sum_{i,j=1}^{n}\lambda {∥f_{i}-f_{j}∥}_{2}^{2}s_{ij}$, the optimal *S* can be obtained from this problem
  (9) $$\begin{array}{cc} \; & \min_{S,P,F}\sum_{i,j=1}^{n}(∥P^{T}x_{i}-P^{T}x_{j}{∥}_{2}^{2}s_{ij}+\gamma s_{ij}^{2})+\frac{\beta }{N_{r}}\sum_{i,j=1}^{n}\sum_{k=1}^{N_{r}}∥P^{T}x_{i}^{k}-P^{T}x_{j}^{k}{∥}_{2}^{2}s_{ij}+\sum_{i,j=1}^{n}\lambda {∥f_{i}-f_{j}∥}_{2}^{2}s_{ij}\\ \; & \forall i,s_{i}^{T}1=1,0\leq s_{i}\leq 1,P^{T}StP=I. \end{array}$$

Let $d_{ij}^{px}={∥P^{T}x_{i}-P^{T}x_{j}∥}_{2}^{2}$, $d_{ij}^{f}={∥f_{i}-f_{j}∥}_{2}^{2}$, and $d_{ij}^{pk}=\frac{1}{N_{r}}\sum_{k=1}^{N_{r}}{∥P^{T}x_{i}^{k}-P^{T}x_{j}^{k}∥}_{2}^{2}$, and denote $d_{ij}^{p}=d_{ij}^{px}+\lambda d_{ij}^{f}+\beta d_{ij}^{pk}$. Finally, the *S* is updated by

(10) $$\begin{array}{c} \min_{s_{i}^{T}1=1,0\leq s_{i}\leq 1}{∥s_{i}+\frac{1}{2\gamma }d_{i}^{p}∥}_{2}^{2}. \end{array}$$

According to [18], the solution of $s_{ij}$ (the ith element of $s_{i}$) to the above problem is $s_{ij}=-\frac{d_{ij}}{2\gamma }$.

### 2.3. Computational Complexity Analysis for PCSSCAG

This subsection briefly analyzes the computational complexity of Algorithm 1. The computational cost of optimal PCSSCAG mainly comes from updating the variables *S*, *P*, and *F*. Without loss of generality, we suppose that the raw data contain *n* samples with *m* features, and the projection *P* reduces the raw into a low-dimensional space with *d* features, where d≪m,n. The complexity of updating *S* in each iteration is $O(nd^{2})$. Updating *P* and *F* require solving two eigenvalue problems, whose complexities are at most $O(n^{3})$, respectively. Thus, the total computational complexity of solving PCSSCAG is at most $O(t\left(nd^{2}+2n^{3}\right))$, where *t* is the number of iterations for Algorithm 1. Since d≪n, the complexity of PCSSCAG is $O(tn^{3})$, which is only highly related to the size of the samples. This implies that the PCSSCAG method can process high-dimensional data effectively.
**Algorithm 1** Optimization Algorithm for Solving PCSSCAG**Input:** Dataset $X\in R^{n\times d}$, cluster number *c*, reduced dimension *m*, parameter γ, λ, and β.**Initialization:**
Initialize *S* by computing the problem (1).**while** not converged **do**1: Update *F* by computing problem (6).2: Update *P* by computing problem (8).3: For each *i*, update the *i*-th row of *S* by computing problem (10).**Output:***S*, *P*


### 2.4. Large-Scale HSI Clustering Strategy with PCSSCAG

**Figure 1:**
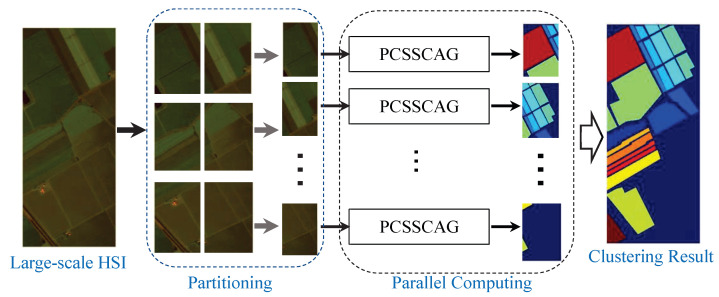
The parallel strategy for large-scale HSI.

As discussed in Section 2.3, the computational complexity of PCSSCAG is highly related to the number of samples. Therefore, PCSSCAG requires more computational cost while clustering large-scale HSIs. To tackle this problem, a parallel computation strategy as shown in Figure 1 is designed to deal with the large-scale HSIs. In this strategy, HSIs are first divided into several small non-overlapping parts. Then, PCSSCAG are parallelly adopted to do clustering on each parts. Finally, the overall clustering result is obtained by combining the clustering results of all parts together. To validate the efficiency for large-scale HSI clustering, experiments are conducted on some benchmark large-scale HSI datasets in the next section.

## 3. Experiments

In this part, the performance of the proposed PCSSCAG is systematically evaluated with several state-of-the-art methods, such as NMF, FCM, FCM_S1, FSCAG, CAN, and PCAN. Specifically, to verify the usefulness of the integrated low-dimensional projection, the PCSSCAG degenerated model (i.e., CSSCAG) is used as a comparative method for the ablation study. To quantitatively evaluate the methods, the used evaluation metrics are the clustering accuracy of each category, overall accuracy (OA), Kappa coefficient (κ), and normalized mutual information (*NMI*). The following is the calculation formula of NMI.

(11) $$\begin{array}{c} NMI\left(X,Y\right)=\frac{2I(X,Y)}{H(X)+H(Y)}, \end{array}$$

where H(X), H(Y) are the respective information entropies of *X* and *Y*, and I(X,Y) is the mutual information of *X* and *Y*. In order to ensure the fairness of the experiment, the parameter values of the comparative methods are adjusted to the optimum. Furthermore, each method is rerun 100 times to eliminate the effect of random initialization, and the average result is reported as the performance evaluation.

### 3.1. Data Description

In the experiments, two different scales of HSIs are utilized to thoroughly validate the effectiveness of the proposed PCSSCAG method. Specifically, the Indian Pines and Salinas-A are two small-scale HSI datasets firstly used for testing the PCSSCAG method. Then, the whole Salinas and University of Pavia datasets are employed for verifying the clustering performance on large-scale HSIs similar to [32,33]. The more detailed descriptions of the datasets are presented in the following.

The Indian Pine dataset was gathered by the Airborne Visible Infrared Imaging (AVIRIS) sensor. The number of bands of the Indian Pines dataset used in our experiment was reduced to 200 by removing bands covering the region of water absorption. In particular, a typical part of the Indian Pines dataset, with the size of 85 × 68 was selected for experiments, which includes four classes: corn-notill, grass-trees, soybean-nottill, and soybean-mintill. The Salinas dataset was acquired by AVIRIS sensor over the Salinas Valley. It includes 224 spectral bands, with the size of 512 × 217. Similar to the Indian Pines scene, 24 water absorption bands were discarded. Salinas-A is a small subscene of Salinas image, with the size of 83 × 86. Salinas-A includes six classes: broccoli-green-weeds-1, corn-senesced-green-weeds, lettuce-romaine-4wk, lettuce-romaine-5wk, lettuce-romaine-6wk and lettuce-romaine-7wk. The University of Pavia dataset was obtained by German airborne reflection optical spectral imager. The size of the dataset is 610 × 340 and with 103 spectral bands. The University of Pavia dataset includes nine classes.

### 3.2. Parameter Analysis

In the proposed methods, there are two parameters (i.e., the size of the sliding window and β) that need to be pre-determined. In our experiments, the size of the sliding window is empirically set as 3×3. For the value of β, we select the value from 0 to 1 at an interval of 0.1 and finally determine the reasonable β value according to the best performance of clustering.

(1) Parameter analysis for the small-scale HSIs

**Figure 2:**
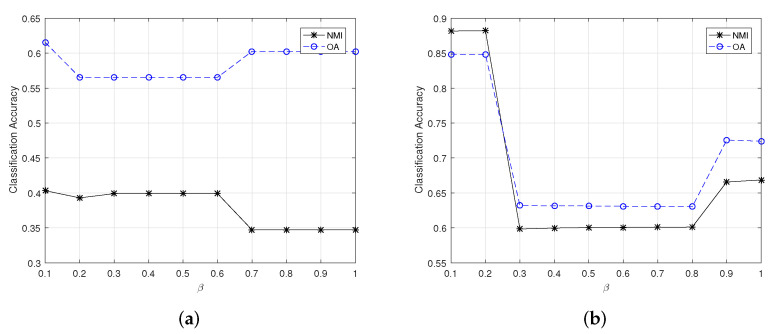
The change of clustering OA and NMI for small-scale HSI datasets yielded by CSSCAG with different β. (**a**) The Indian Pine dataset, (**b**) the Salinas-A dataset.

**Figure 3:**
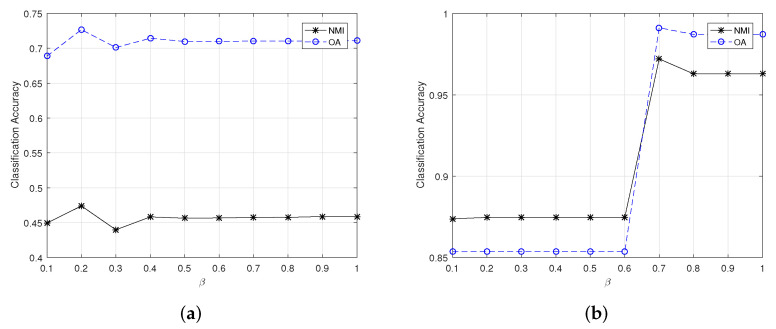
The change of clustering OA and NMI for small-scale HSI datasets yielded by PCSSCAG with different β. (**a**) The Indian Pine dataset, (**b**) the Salinas-A dataset.

Figure 2 and Figure 3 show the changes of clustering OA and NMI for small-scale HSI datasets yielded by CSSCAG and PCSSCAG, respectively. From Figure 3, it is easy to see that the optimal value of β in CSSCAG is 0.1 for the Indian Pine dataset and 0.2 for Salinas-A. Likewise, the suitable value of β for Indian Pine and Salinas-A datasets can be respectively set as 0.2 and 0.7 in PCSSCAG.

(2) Parameter analysis for the large-scale HSIs

**Figure 4:**
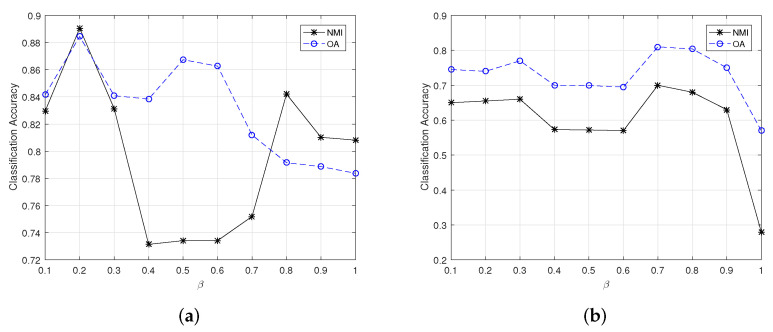
The change of clustering OA and NMI for large-scale HSIs yielded by CSSCAG with different β. (**a**) The Salinas dataset, (**b**) The University of Pavia dataset.

**Figure 5:**
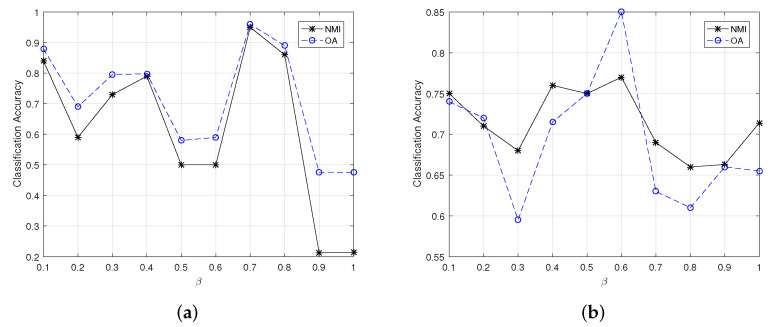
The change of clustering OA and NMI for large-scale HSIs yielded by PCSSCAG with different β. (**a**) The Salinas dataset, (**b**) the University of Pavia dataset.

The changes of clustering OA and NMI for the large-scale HSI datasets are exhibited in Figure 4 and Figure 5. It can be learned that the most appropriate value of β for the University of Pavia dataset in CSSCAG and PCSSCAG are 0.7 and 0.6, respectively. For the Salinas datasets, the suitable β is same as the Salinas-A dataset.

### 3.3. Experimental Results and Analysis

(1) Clustering for small-scale HSIs

The first experiment is conducted on the two small-scale HSI datasets. The clustering accuracy of each category, OA, NMI, and κ of the Indian Pines and Salinas-A datasets yielded by different clustering algorithms are listed in Table 1 and Table 2. From Table 1 and Table 2, it can be seen that the OA, NMI, and κ obtained by the classical methods (i.e., NMF, FCM, and FCM_S1) are relatively low for the two small-scale HSIs. It is obvious that the graph-based methods (i.e., CAN, PCAN, FCAG, CSSCAG, and PCSSCAG) perform better than the classical clustering methods. Among the graph-based methods, the CSSCAG and PCSSCAG methods that with spatial-spectral constraint obtain better clustering performance than CAN and PCAN, respectively. More importantly, the proposed PCSSCAG achieves the highest clustering accuracy. Comparing with the other methods, PCSSCAG yields increases of more than 4 percent in OA and 3 percent in κ on the Indian Pines dataset, respectively. PCSSCAG achieves great improvement on Salinas-A in clustering accuracy and presents the highest clustering accuracy with OA of 0.99, NMI of 0.97, and κ of 0.99.

**Figure 6:**
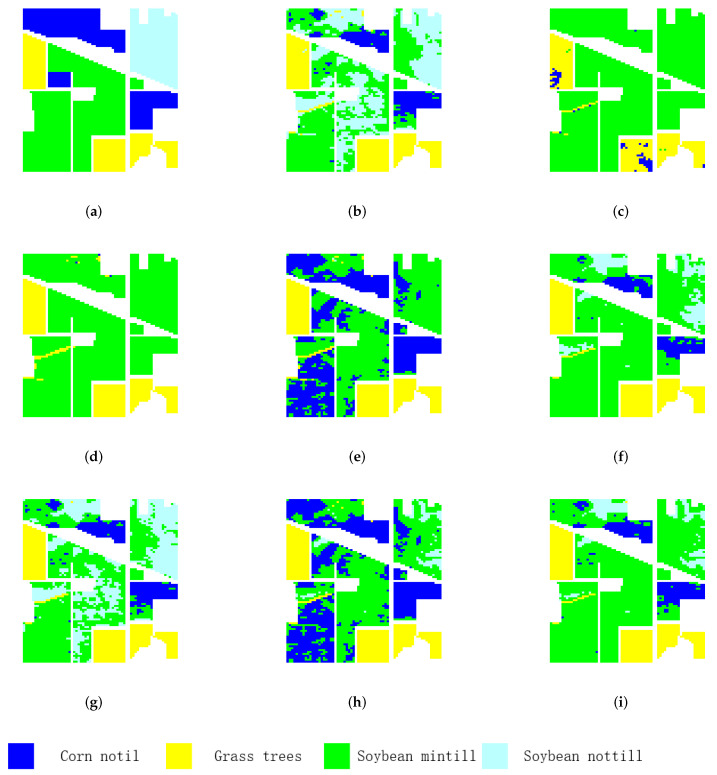
Cluster maps of different methods for the Indian Pines dataset. (**a**) Ground truth, (**b**) NMF, (**c**) FCM, (**d**) FCM_S1, (**e**) CAN, (**f**) PCAN, (**g**) FSCAG, (**h**) CSSCAG, and (**i**) PCSSCAG.

**Figure 7:**
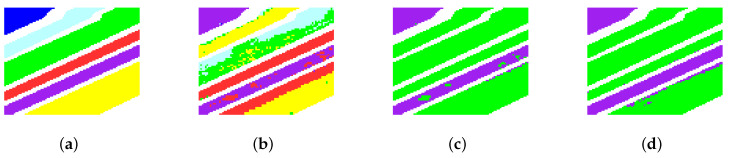

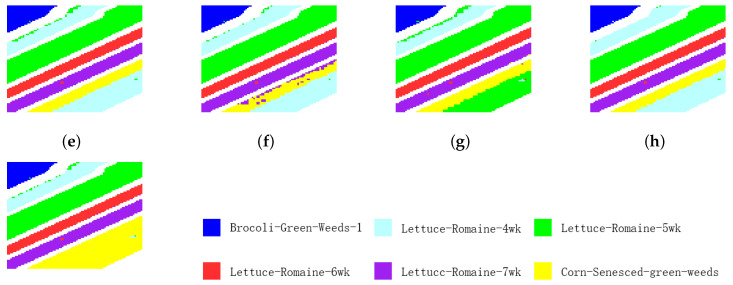
Cluster maps of different methods for the the Salinas-A. (**a**) Ground truth, (**b**) NMF, (**c**) FCM, (**d**) FCM_S1, (**e**) CAN, (**f**) PCAN, (**g**) FSCAG, (**h**) CSSCAG, and (**i**) PCSSCAG.

Figure 6 and Figure 7 show the corresponding cluster maps of different clustering methods for Indian Pines and Salinas-A, respectively. From the figures, a consistent conclusion can be learned from the cluster maps. It can be found that there are almost only two clusters in the cluster maps of Indian Pines yielded by FCM and FCM_S1. They failed to divide some similar classes, such as soybean-mintill and soybean-nottill. FCM and FCM_S1 also obtain similar results on Salinas-A. The graph-based clustering methods perform better cluster maps for both small-scale datasets. In particular, PCSSCAG products a more smooth clustering map than all comparative methods.

(2) Clustering for large-scale HSIs

**Figure 8:**
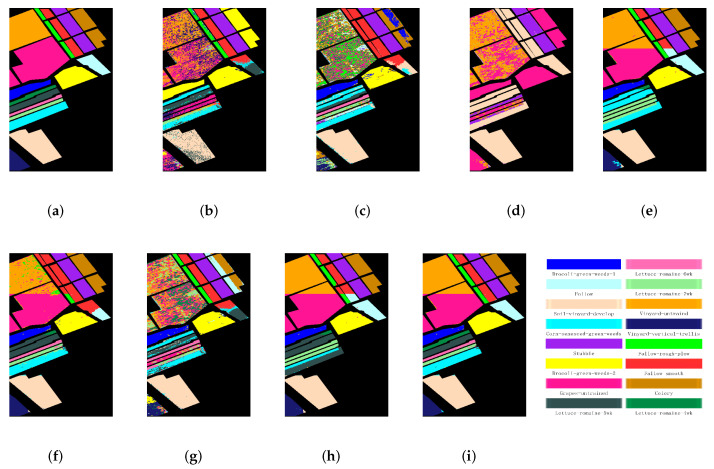
Cluster maps of different methods for the Salinas dataset. (**a**) Ground truth, (**b**) NMF, (**c**) FCM, (**d**) FCM_S1, (**e**) CAN, (**f**) PCAN, (**g**) FSCAG, (**h**) CSSCAG, and (**i**) PCSSCAG.

The second experiment will validate the performance of the proposed PCSSCAG with two large-scale HSIs, i.e., Salinas and University of Pavia. Those two large-scale HSI datasets include quite a lot classes. Particularly, they contain more complex land-cover classes and the spectral signatures of some classes are very similar, which results in a more challenging clustering task. The quantitative evaluation and visual clustering results of the Salinas dataset are reported in Table 3 and Figure 8. From Table 3, it is obvious that the conventional clustering methods, such as NMF and FCM, achieve competitive clustering performance. However, the accuracy of some categories is still unsatisfactory. Comparing to NMF, FCM, and FCM_S1, the graph-based methods (i.e., CAN, PCAN, FSCAG, CSSCAG, and PCSSCAG) yield better clustering results. Specifically, compared to FCM and NMF, PCAN achieves an improvement of over 3 percent in OA and κ, and almost 2 percent in NMI. CSSCAG and PCSSCAG obtain higher clustering accuracy than CAN and PCAN, respectively. That implies the spatial-spectral constraint can effectively improve the cluster performance of HSIs. PCSSCAG improves the clustering performance by integrating a projection to reduce the redundancy while comparing to CSSCAG. More importantly, the proposed PCSSCAG method achieves the best clustering result. It is not hard to find that the clustering maps in Figure 8 show a consistent results with the accuracy in Table 3.

**Figure 9:**
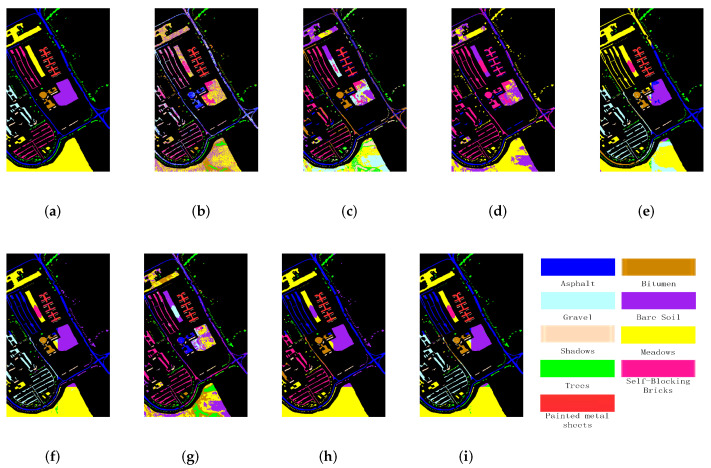
Cluster maps of different methods for the University of Pavia dataset. (**a**) Ground truth, (**b**) NMF, (**c**) FCM, (**d**) FCM_S1, (**e**) CAN, (**f**) PCAN, (**g**) FSCAG, (**h**)CSSCAG, and (**i**) PCSSCAG.

The experimental results of the University of Pavia dataset are shown in Table 4 and Figure 9. It can be seen from the Table 4 that the clustering results of the classical clustering methods (such as NMF, FCM, and FCM_S1) are very poor. For instance, the OA of FCM and NMF is less than 0.5 and samples from several categories failed to be divided by FCM_S1. Comparing with the classical clustering methods, the graph-based methods achieve better clustering performance. In particular, PCSSCAG achieves the highest clustering accuracy, which outperforms the comparative methods more than 4 percent in OA, 6 percent in NMI, and 4 percent in κ, respectively. A similar conclusion can be obtained from the cluster maps in Figure 9 for the University of Pavia dataset. From the above experiments, it can be found that the proposed methods greatly improve the performance of clustering through the incorporation of spatial-spectral constraint and projection learning. Those experimental results on both large-scale datasets validate that the clustering performance of PCSSCAG is still acceptable for large-scale HSI analysis via the designed parallel computing strategy.

Overall, from the experimental results two different scales of HSI datasets, it can obtain that the graph-based methods perform better clustering results than the other comparative methods. Among the graph-based methods, the proposed PCSSCAG method achieves the best performance in both quantitative and visual results. Specifically, comparing with CAN and PCAN, the spatial-spectral constraint imposed on PCSSCAG helps to improve the clustering accuracy by simultaneously making full use of the spatial and spectral information of HSI data. The ablation study (i.e., comparing PCSSCAG with CSSCAG) demonstrates that the low-dimensional projection integrated in PCSSCAG can reduce the redundancy to improve the effectiveness and avoid the curse of dimensionality problem.

## 4. Conclusions

In this paper, a clustering method with a spatial–spectral constrained adaptive graph is proposed for HSI clustering. The proposed method first utilizes both spectral and spatial information of HSI data to construct a more precise adjacency graph, which helps to enhance the robustness against noise. Then, projection learning is employed to alleviate the negative influences caused by the high dimensionality, which further improved the accuracy of clustering. At last, extensive experiments are conducted on several real hyperspectral datasets to verify the proposed method, and the experimental results show that the proposed PCSSCAG method performs better than all of the involved comparative methods.

However, the weight matrix associated to the adjacency graph in the proposed method is highly related to the size of samples, which requires more memory while clustering large-scale HSIs. The designed parallel strategy for large-scale HSIs fails to consider the correlation among patches, which may result in some loss of valuable global information. In further studies, we will make an effort to develop multiple graphs based clustering method for large-scale HSI processing.

## Figures and Tables

**Table 1:** Clustering accuracy of each category, OA, NMI, and κ of different clustering methods on Indian Pines dataset.

Class	Method							
	NMF	FCM	FCM_S1	CAN	PCAN	FSCAG	CSSCAG	PCSSCAG
corn-notil	0.41	65	0	0.72	0.43	0.34	0.72	0.47
Grass-trees	0.87	0.93	1	1	1	1	1	1
Soybean-mintill	0.49	0.42	0.09	0	0.29	0.64	0.16	0.22
Soybean-nottill	0.68	0.31	0.96	0.60	0.95	0.71	0.59	0.95
OA	0.62	0.51	0.59	0.59	0.69	0.69	0.62	0.73
NMI	0.38	0.37	0.32	0.39	0.39	0.47	0.40	0.47
κ	0.41	0.32	0.33	0.41	0.55	0.55	0.45	0.58

**Table 2:** Clustering accuracy of each category, OA, NMI, and κ of different clustering methods on Salinas-A.

Class	Method							
	NMF	FCM	FCM_S1	CAN	PCAN	FSCAG	CSSCAG	PCSSCAG
Brocoli-green-weeds-1	0.72	0.99	0	1	1	0.99	1	1
Corn-senesced-green-weeds	0.49	0.34	0	0.41	0.36	0.43	0.40	1
Lettuce-romaine-4wk	0.27	0.69	0	0.91	0.97	0.87	1	0.95
Lettuce-romaine-5wk	0.83	0.64	1	1	1	1	1	1
Lettucc-romaine-6wk	0.60	1	0	0.99	0.99	1	0.99	0.99
Lettucc-romaine-7wk	0.95	0.94	1	0.99	1	0.99	1	1
OA	0.65	0.69	0.58	0.84	0.83	0.83	0.85	0.99
NMI	0.61	0.67	0.51	0.86	0.84	0.78	0.88	0.97
κ	0.69	0.63	0.48	0.8	0.8	0.79	0.81	0.99

**Table 3:** Clustering accuracy of each category, OA, NMI, and κ of different clustering methods on Salinas dataset.

Class	Method							
	NMF	FCM	FCM_S1	CAN	PCAN	FSCAG	CSSCAG	PSSCAG
Brocoli-green-weeds-1	0.37	0.99	0	0.99	0.99	0.98	1	0.99
Brocoli-green-weeds-2	0.72	0.88	1	1	0.99	0.97	1	
Follow	0.11	0	0	1	0.43	0	1	1
Fallow-rough-plow	0.47	0.1	0	0.99	0.2	0.98	0.85	1
Fallow-smooth	0.72	0.99	0	0.81	1	1	0.81	0.98
Stubble	0.93	0.91	0.97	1	1	1	1	1
Celery	0.56	0.61	0	1	1	0.53	1	1
Grapes-untrained	0.54	0.32	0.61	0.85	0.85	0.36	0.85	0.85
Soil-vinyard-develop	0.77	0.97	1	1	0.99	0.98	1	0.99
Corn-senesced-green-weeds	0.40	0.39	0	0.71	0.88	0.51	0.28	0.98
Lettuce-romaine-4wk	0.14	0.19	0	0.18	0.18	0	0.18	1
Lettuce-romaine-5wk	0.31	0.58	0	0.01	1	0.80	1	1
Lettucc-romaine-6wk	0.49	0.98	0	0	0.97	0.98	0	0.97
Lettucc-romaine-7wk	0.40	0.8	0	0.99	0.94	0.15	0.99	0.93
Vinyard-untraind	0.40	0.34	0	0.99	0.9	0.40	0.98	0.99
Vinyard-vertical-trellis	0.39	0.42	0	0.92	0.99	0.64	0.98	0.99
OA	0.55	0.56	0.38	0.87	0.89	0.62	0.88	0.96
NMI	0.62	0.67	0.39	0.88	0.88	0.72	0.89	0.95
κ	0.53	0.52	0.3	0.85	0.87	0.59	0.86	0.96

**Table 4:** Clustering accuracy of each category, OA, NMI, and κ of different clustering methods on the University of Pavia dataset.

Class	Method							
	NMF	FCM	FCM_S1	CAN	PCAN	FSCAG	CSSCAG	PCSSCAG
Asphalt	0.11	0.64	1	0.47	0.93	0.63	0.91	0.91
Meadows	0.65	0.29	0.97	0.81	0.94	0.34	0.95	0.94
Gravel	0.71	0	0	0.93	0.93	0.01	0	0.95
Trees	0.25	0.49	0	0.87	0.43	0.84	0.31	0.86
Painted metal sheets	0.12	0.78	0	1	0.86	0.98	0.99	1
Bare Soil	0.71	0.33	0	0.85	0.92	0.39	0.95	0.92
Bitumen	0.58	0.68	0	0.85	0.85	0	0.92	0.89
Self-Blocking Bricks	0.58	0.77	0	0	0	0.91	0.52	0
Shadows	0.50	0	0	0.81	0.8	0.93	0.83	0.83
OA	0.49	0.42	0.56	0.71	0.81	0.50	0.81	0.85
NMI	0.44	0.45	0.27	0.57	0.71	0.51	0.70	0.77
κ	0.44	0.34	0.35	0.61	0.74	0.39	0.75	0.79

## Data Availability

The hyperspectral datasets used in this study are openly available at https://www.ehu.eus/ccwintco/index.php?title=Hyperspectral_Remote_Sensing_Scenes.

## References

[1] Tong Q., Xue Y., Zhang L. (2014). Progress in hyperspectral remote sensing science and technology in China over the past three decades. IEEE J. Sel. Top. Appl. Earth Obs. Remote Sens..

[2] Qu J.H., Xu Y.S., Dong W.Q., Li Y.S., Du Q. (2022). Dual-branch difference amplification graph convolutional network for hyperspectral image change detection. IEEE Trans. Geosci. Remote Sens..

[3] Li H., Jia S., Le Z. (2019). Quantitative analysis of soil total nitrogen using hyperspectral imaging technology with extreme learning machine. Sensors.

[4] Yuan J., Wang S., Wu C., Xu Y. (2022). Fine-grained classification of urban functional zones and landscape pattern analysis using hyperspectral satellite imagery: A case study of wuhan. IEEE J. Sel. Top. Appl. Earth Obs. Remote Sens.

[5] Stuart M.B., Davies M., Hobbs M.J., Pering T.D., McGonigle A.J.S., Willmott J.R. (2022). High-resolution hyperspectral imaging using low-cost components: Application within environmental monitoring scenarios. Sensors.

[6] Zhao C., Zhao H., Wang G., Chen H. (2020). Improvement SVM classification performance of hyperspectral image using chaotic sequences in artificial bee colony. IEEE Access..

[7] Tang Y.F., Li X.M., Xu Y., Liu Y., Wang J.Z., Liu C.Y., Liu S.C. Hyperspectral image classification using sparse representation-based classifier. Proceedings of the IEEE Geoscience and Remote Sensing Symposium.

[8] Liu X., Hu Q., Cai Y., Cai Z. (2020). Extreme learning machine-based ensemble transfer learning for hyperspectral image classification. IEEE J. Sel. Top. Appl. Earth Observ. Remote Sens..

[9] Zhang Y., Liu K., Dong Y., Wu K., Hu X. (2020). Semisupervised classification based on SLIC segmentation for hyperspectral image. IEEE Geosci. Remote Sens. Lett..

[10] Hartigan J.A., Wong M.A. (1979). A k-means clustering algorithm. Appl. Stat..

[11] Jin Y., Ding L., Yang F., Qian L., Zhi C. LoRa network planning based on improved ISODATA algorithm. Proceedings of the International Conference on Wireless Communications and Signal Processing (WCSP).

[12] Dong L., Yuan Y., Luxs X. (2021). Spectral–spatial joint sparse NMF for hyperspectral unmixing. IEEE Trans. Geosci. Remote Sens..

[13] Huo H., Guo J., Li Z.L. (2018). Hyperspectral image classification for land cover based on an improved interval typ-II fuzzy c-means approach. Sensors.

[14] Ahmed M.N., Yamany S.M., Mohamed N.A., Farag A., Moriarty T. (2002). A modified fuzzy c-means algorithm for bias field estimation and segmentation of MRI data. IEEE Trans. Med. Imaging.

[15] Chen S., Zhang D. (2004). Robust image segmentation using FCM withspatial constraints based on new kernel-induced distance measure. IEEE Trans. Syst. Man Cybern..

[16] Li C.H., Huang W.C., Kuo B.C., Hung C.C. (2008). A novel fuzzy weighted C-means method for image classification. Int. J. Fuzzy Syst..

[17] Hung C., Kulkarni S., Kuo B. (2011). A new weighted fuzzy C-Means clustering algorithm for remotely sensed image classification. IEEE J. Sel. Top. Signal Process..

[18] Krinidis S., Chatzis V. (2010). A robust fuzzy local information C-means clustering algorithm. IEEE Trans. Image Process..

[19] Zhang H., Wang Q., Shi W., Hao M. (2017). A novel adaptive fuzzy local information *C* -Means clustering algorithm for remotely sensed imagery classification. IEEE Trans. Geosci. Remote Sens.

[20] Zhai H., Zhang H., Li P., Zhang L. (2021). Hyperspectral image clustering: Current achievements and future lines. IEEE Geosci. Remote Sens. Mag..

[21] Elhamifar E., Vidar R. (2013). Sparse subspace clustering: Algorithm, theory, and application. IEEE Trans. Pattern Anal. Mach. Intell..

[22] Elhamifar E., Vidar R. Sparse subspace clustering. Proceedings of the IEEE Conference on Computer Vision and Pattern Recognition.

[23] Zhang H., Zhai H., Zhang L., Li P. (2016). Spectral–spatial sparse subspace clustering for hyperspectral remote sensing images. IEEE Trans. Geosci. Remote Sens..

[24] Li A., Qin A., Shang Z., Tang Y.Y. (2019). Spectral-spatial sparse subspace clustering based on three-dimensional edge-preserving filtering for hyperspectral image. Int. J. Pattern Recognit. Artif. Intell..

[25] Huang S., Zhang H., Pižurica A. (2019). Semisupervised sparse subspace clustering method with a joint sparsity constraint for hyperspectral remote sensing images. IEEE J. Sel. Top. Appl. Earth Observ. Remote Sens..

[26] Zeng M., Cai Y., Liu X., Cai Z., Li X. Spectral-spatial clustering of hyperspectral image based on Laplacian regularized deep subspace clustering. Proceedings of the IEEE International Geoscience and Remote Sensing Symposium.

[27] Lei J., Li X., Peng B., Fang L., Ling N., Huang Q. (2021). Deep spatial-spectral subspace clustering for hyperspectral image. IEEE Trans. Circuits Syst. Video Technol..

[28] Nie F., Wang X., Huang H. Clustering and projected clustering with adaptive neighbors. Proceedings of the 20th ACM International Conference on Knowledge Discovery and Data Mining.

[29] Wang R., Nie F., Yu W. (2017). Fast spectral clustering with anchor graph for large hyperspectral images. IEEE Geosci. Remote Sens. Lett..

[30] Wang R., Nie F., Wang Z., He F., Li X. (2019). Scalable graph-based clustering with nonnegative relaxation for large hyperspectral image. IEEE Trans. Geosci. Remote Sens..

[31] Peng J., Sun W., Li H.C., Li W., Meng X., Ge C., Du Q. (2022). Low-rank and sparse representation for hyperspectral image processing: A review. IEEE Geosci. Remote Sens. Mag..

[32] Yang X., Xu Y., Li S., Liu Y., Liu Y. (2022). Fuzzy embedded clustering based on bipartite graph for large-scale hyperspectral image. IEEE Geosci. Remote Sens. Lett..

[33] Zhai H., Zhang H., Zhang L., Li P. (2021). Sparsity-based clustering for large hyperspectral remote sensing images. IEEE Trans. Geosci. Remote Sens..

